# Simulation of the Response of the Inner Hair Cell Stereocilia Bundle to an Acoustical Stimulus

**DOI:** 10.1371/journal.pone.0018161

**Published:** 2011-03-31

**Authors:** Sonya T. Smith, Richard S. Chadwick

**Affiliations:** 1 Department of Mechanical Engineering, Howard University, Washington, D.C., United States of America; 2 Section on Auditory Mechanics, National Institute on Deafness and other Communication Disorders, National Institutes of Health, Bethesda, Maryland, United States of America; University of Maribor, Slovenia

## Abstract

Mammalian hearing relies on a cochlear hydrodynamic sensor embodied in the inner hair cell stereocilia bundle. It is presumed that acoustical stimuli induce a fluid shear-driven motion between the tectorial membrane and the reticular lamina to deflect the bundle. It is hypothesized that ion channels are opened by molecular gates that sense tension in tip-links, which connect adjacent stepped rows of stereocilia. Yet almost nothing is known about how the fluid and bundle interact. Here we show using our microfluidics model how each row of stereocilia and their associated tip links and gates move in response to an acoustical input that induces an orbital motion of the reticular lamina. The model confirms the crucial role of the positioning of the tectorial membrane in hearing, and explains how this membrane amplifies and synchronizes the timing of peak tension in the tip links. Both stereocilia rotation and length change are needed for synchronization of peak tip link tension. Stereocilia length change occurs in response to accelerations perpendicular to the oscillatory fluid shear flow. Simulations indicate that nanovortices form between rows to facilitate diffusion of ions into channels, showing how nature has devised a way to solve the diffusive mixing problem that persists in engineered microfluidic devices.

## Introduction

**Figure 1:**
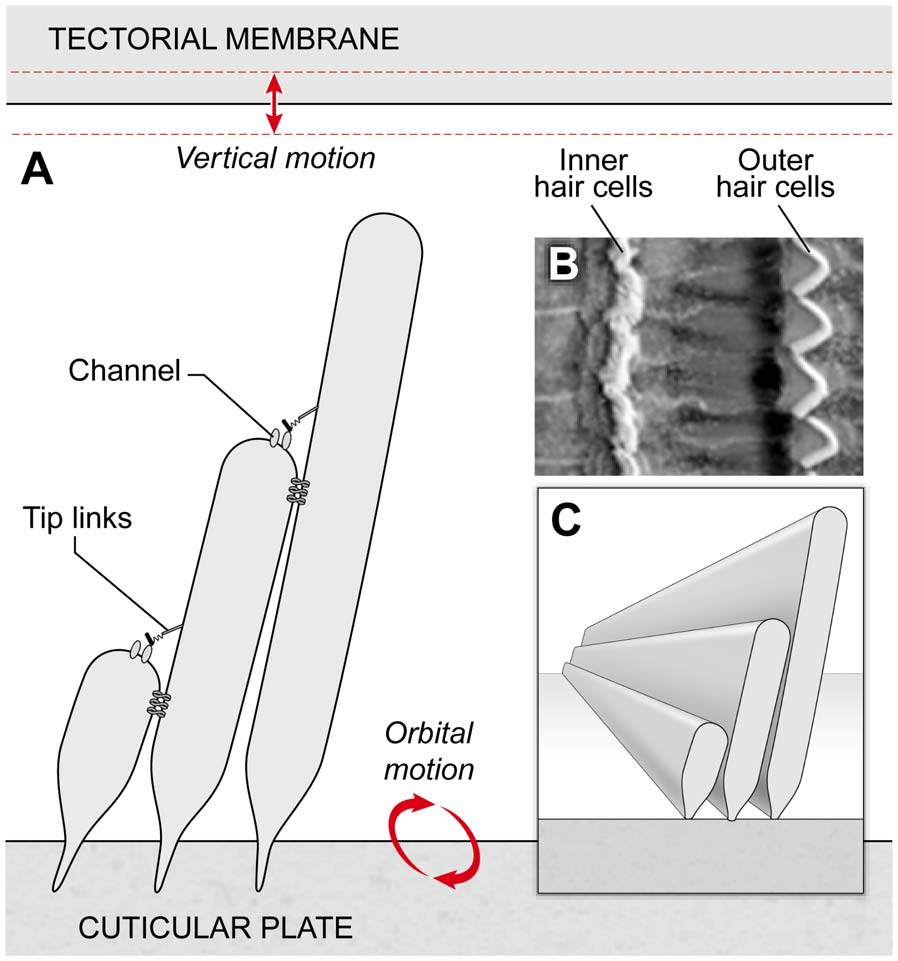
Model inner hair cell bundle. The orbital motion of the lower boundary, the reticular lamina (the cuticular plate is part of the reticular lamina) and the vertical oscillatory motion of upper boundary (tectorial membrane) hydrodynamically drive the bundle. Three stepped rows of stereocilia (actin-filled rod structures) are connected by two sets of tip links with gating springs and six horizontal top connectors. Upper tip links connect the tallest and middle rows; lower tip links connect the shortest and middle rows. All elements are assumed to be elastic with bending and stretching energies. The fluid is viscous and incompressible.

The inner hair cell stereocilia bundle performs the role of transducer during the process of mammalian hearing. Acoustic stimuli deflect the hair bundle to open ion channels, resulting in cation influx and the subsequent release of a neurotransmitter at the base of the cell [1], [2]. Hypotheses for this transduction include fluid shear-driven motion between the tectorial membrane and the reticular lamina to deflect the bundle [3], [4]. It is presumed that ‘molecular gates’ sense tension in tip-links that connect adjacent stepped rows of stereocilia to open the channels [5]. The simplest hypothesis for the deformation of the hair bundle, either by a mechanical probe or from fluid motion, is that each stereocilium rotates as a rigid rod about its insertion into the cuticular plate (Fig. 1). Equal rotations of the three rows of stereocilia then imply that the tip-link/gate/membrane complex would undergo a fractional length change. This simple model is appealing since it tends to synchronize ion channel gate openings and thus increase hearing sensitivity. But once stereocilia are allowed to deflect in the presence of fluid shear, which itself is altered by the presence of the hair bundle, the stereocilia will splay, and the fractional length changes of upper and lower tip-links may lose synchronization. The distance between the top of the tallest row of stereocilia and the bottom of the tectorial membrane turns out to control the amount of splay. When a mechanical probe is used to deflect the bundle and fluid shear is not present, splay may also be controlled by top horizontal connectors and sliding adhesion [6].

In the spirit of Occam's razor, we should look at the next simplest model to explain the interaction of the fluid with the bundle. To that end, we note that inner hair cell stereocilia are arranged in nearly straight rows to form a continuous fence-like structure compared to the V-shaped or W-shaped patterns seen from outer hair cells (Fig. 1B). We also note that gaps between individual stereocilium are small compared to the gap between the tallest stereocilium and the underside of the tectorial membrane (100 nm vs.1000 nm). Also, in many preparations, the spacing between adjacent stereocilia in neighboring cells is similar to the spacing between adjacent stereocilia on the same cell.

This geometry suggests the dominant flow will be over the bundle, rather than around individual stereocilium. This simplification allows us to model the flow and bundle in 2D rather than 3D (Fig. 1C), enabling us to increase the resolution in the model. The inner hair cell bundle model shown in Fig. 1A and 1C is driven by fluid motion resulting from the orbital oscillatory motion of the reticular lamina reported from an acoustically driven preparation [7]. It is important to notice that the orbital motion has both horizontal and vertical components. The upper boundary, the tectorial membrane, is assumed to be stationary in the horizontal direction to provide an oscillatory shear stimulus, and have the identical vertical direction motion as the reticular lamina so that the vertical distance between the two boundaries is unchanged during their motions. Stereocilia, tip-links, gating springs located at the lower ends of the tip-links [8], and horizontal links are all treated as elements possessing both stretching and flexural elastic energies. The hair bundle-fluid interaction computation is performed using the immersed boundary method [9]. Our code was designed to capture nanometer-sized motions in a micron-sized domain.

## Results and Discussion

### Calibration of model

The model was dynamically calibrated at 200 Hz and 98 dB sound pressure level to match the reported horizontal motion (amplitude and phase) of the tallest stereocilia row reported previously [7], Fig. S1. This involved adjusting geometric and elastic parameters, resulting in values shown in Table S1.

### Synchronization of upper and lower tip link peak tension by the tectorial membrane

**Figure 2:**
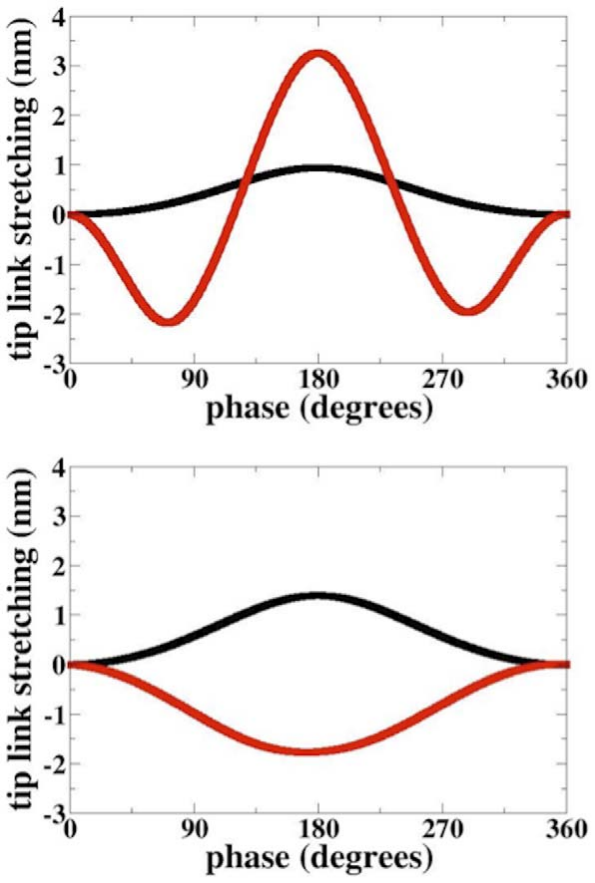
Tip link stretching as a function of phase of reticular lamina motion. Upper panel: tectorial membrane in normal position, red-upper tip link; black- lower tip link. Lower panel; the tectorial membrane-reticular lamina spacing is widened by 5 microns; same color code. Molecular gate does not open when tip link is in compression (negative values of stretching). Model confirms the critical role of the tectorial membrane for hearing sensitivity.

The key results in Fig. 2 are the phasic behavior of the upper and lower tip link nanometer-sized length changes resulting from the orbital motion of the reticular lamina. With the tectorial membrane in the normal position, both upper and lower tip links have maximal positive stretches synchronized at a phase of 180 degrees, which corresponds to the reticular lamina being displaced maximally to the right and downward. When the vertical distance between the tectorial membrane and reticular lamina is changed from 5 to 10 microns, keeping everything else the same, the upper tip link is always in compression (negative length changes), hence its gate will never open. This is consistent with the increased hearing threshold reported in *Tecta* heterozygous mice having an altered tectorial membrane position relative to the reticular lamina [10]. The vertical motion of the reticular lamina is also critical. If the model is excited with horizontal motion only with the tectorial membrane in its normal location, then the lower tip link doesn't develop tension, Fig. S2.

### Predicted motion of the three stereocilia rows

**Figure 3:**
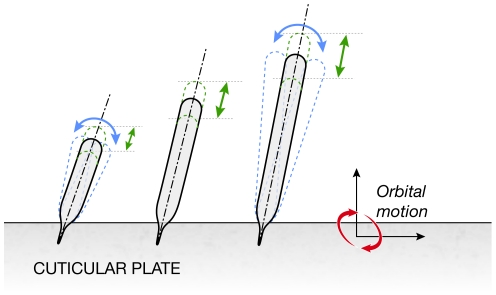
Motion of individual rows of stereocilia. All rows undergo a length change and a rotation, except the middle row has a negligible rotation (see Video S1).

The motion of the individual stereocilia rows corresponding to the tip link stretching patterns of Fig. 2 was not anticipated. The movements are depicted in Fig. 3 and the Video S1. The motions, relative to the reticular lamina, are greatly exaggerated, but have the correct phasic behavior. The smallest and tallest rows rotate as expected, but the middle row does not; instead it changes its length, as do all the rows. The average length change of all the rows was ∼10 nm. This length change is consistent with a longitudinal elastic wave propagating along a rod with a free end, where the displacement of the free end is twice the vertical displacement of the forced end (the reticular lamina). In hindsight, it makes sense that the middle row doesn't rotate since it is shielded from the fluid shear generated by the horizontal motion of the reticular lamina by the shortest and tallest rows. If it did rotate more energy would be dissipated in the endolymph, and the process would be less efficient.

### Formation of a nanovortex aids mixing

**Figure 4:**
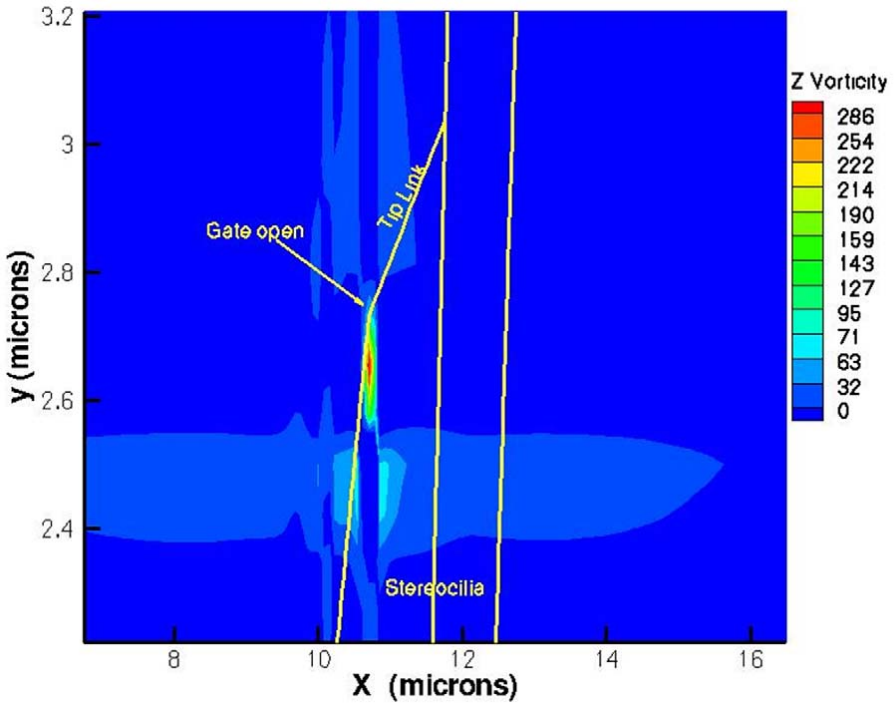
A nanovortex forms near an open gate to augment supply of cations. Closed vorticity contours imply the presence of a fluid eddy. Vorticity values are sec^−1^. Yellow lines show the locations of the stereocilia when the phase of the reticular lamina orbital motion is 214.6 degrees.

The sudden elongation of the gating spring boundary generates the nanovortex, a sub-micron sized eddy seen in Fig. 4. In the model, the gating spring is a 5 nm extension of the tip link that is added to the tip link length when its tension reaches a threshold 26.5 nN. Like an oar in water, vorticity is generated when a boundary moves suddenly. This effect was calculated first by Rayleigh [11]. Indeed the vortices may alleviate a diffusive mixing problem that appears to exist for Ca^++^ ions, which have a concentration of only 20 µM in cochlear endolymph. The number of Ca^++^ ions entering the bundle can be estimated ∼ 10^6^/sec based on a typical total transduction current of 500 pA [12], and the fact that most of the current is due to K^+^ at 160 mM. But diffusion alone can supply Ca^++^ to the bundle only at a rate ∼10^4^/sec based on the estimate D/l^2^, where l is the tip link length (170 nm) and D is the Ca^++^ diffusivity (4×10^−6^ cm^2^/sec) [8]. Thus diffusion alone appears to be unable to supply enough Ca^++^ at the required rate. The vortices can boost the supply of Ca^++^ by convection if the time scale associated with vortical rotation is comparable to the diffusive time scale l^2^/D. From Rayleigh's solution, the vorticity generated at the elongating gating spring is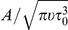, with A the increase in spring length, ν the kinematic viscosity and τ_o_ the elongation time. Taking A = 5 nm, D = 4×10^−6^ cm^2^/s, ν = 0.7 10^−2^ cm^2^/s the vortices augment diffusion if τ_o_ ∼ microsecond or less. This time scale is an order of magnitude smaller than an estimate based on the ability of a bat to hear a 100 kHz signal. Thus it seems that nature has devised a way to solve the diffusive mixing problem that persists in engineered microfluidic devices by using nanovorticies to augment diffusion.

## Materials and Methods

### Fluid-Structure Interaction

The fluid-force from endolymph deforms the stereocilia in the bundle thereby changing the tension in the tip links and initiating channel gating. In return, the stereocilia in the bundle exert forces on the surrounding fluid; altering the flow pattern from one that would exist in the absence of the bundle. The governing equations that account for these interactions are from Peskin [9]:
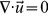
(1a)

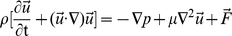
(1b)

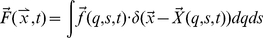
(1c)

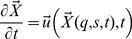
(1d)

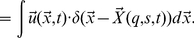
(1e)


Equations (1a) and (1b) are the Navier-Stokes equations for incompressible flow; *p* and 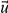 are pressure and velocity, while *ρ* and *µ* are respectively the fluid density and viscosity. In equation (1c) 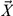 is the Lagrangian variable that describes the position in curvilinear coordinates (*q,s*) of each element of the stereocilia bundle, including tip links and horizontal links. Equation (1d) imposes the no-slip boundary condition on each element of the IHC bundle so that fluid particles on the stereocilia bundle move at the velocity if its elements. The no-slip condition translated to the IHC bundle coordinate system is expressed in equation (1e). Equation (1c) translates 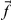, the IHC bundle force per unit volume, onto the fluid grid to facilitate calculation of the last term in the momentum equation (1b),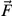, a force per unit volume. This external forcing term accounts for the influence of the bundle on the surrounding fluid.

The force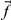 includes contributions of bundle bending and stretching, modified from Stockie & Green [13]. It can be defined as the gradient of an elastic energy per unit volume *E* as follows:
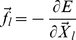
(2a)

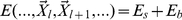
(2b)

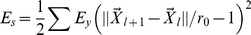
(2c)

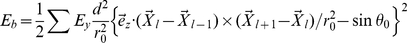
(2d)


In equations [2], r_0_ is the 75 nm resting length between points of the Lagrangian grid, *d* is the local diameter of an elastic element, θ_0_ is the initial external angle between three consecutive grid points (e.g. 0° for a triad without initial curvature), and 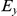 is the effective Young's modulus, 2.3 GPa, for F-actin, based on measurements of Gittes et al. [14]. The sum is over all the discrete Lagrangian elements comprising a moving boundary. The geometric and physical properties of the model are listed in Table S1. We define the base of a stereocilium as one-third of its total height and assume a linear taper of the diameter within this range. The stiffness of links has been assigned a value of 5×10^−4^ N/m estimated by Howard & Hudspeth [15] as the gating stiffness.

### Numerical method

The computational domain is a rectangle 5 microns high by 20 microns long. The height corresponds to the subtectorial gap at the apex of the guinea pig cochlea (the low frequency end). A sensitivity analysis ensured that the domain length was sufficiently long so as not to not affect the results. This fluid domain is divided into a rectangular grid with nodes every 78 nanometers along the length and height. Situated in the middle of the domain is a three-row stereocilia bundle with tip links and horizontal links. The length of the tallest row was chosen so that its clearance from the underside of the tectorial membrane (top boundary) is 0.5 microns. The lengths of the remaining rows in the bundle were chosen so that their height relative to that of the tallest row were similar to those reported in Hackney and Furness [16]. The stereocilia are represented as line forces in the fluid, and the strength of the line forces depends on local bending and stretching energy density. These forces in turn alter the fluid motion to convect the stereocilia rows to updated locations. The power of this method is that it can resolve the motion of the IHC bundle, including the separate rows of stereocilia, along with the endolymphatic fluid motion by decoupling the fluid solver from the solver for the motion of the bundle. This feature provides a significant decrease in computational cost. Another advantage of the immersed boundary method is that the Navier-Stokes equations are solved on a rectangular grid allowing a fast flow solver to be used. In this case the governing equations for the fluid are discretized using finite differences. The time step was 1/1000th of the period of the 200 Hz input frequency. The flow is assumed to start from rest. The sequence of the solution advancement begins by calculating the force densities at the hair-bundle grid points and then distributing them onto the fluid grid of uniform spacing, with *h* = 78 nanometers using the following discrete approximation of the Dirac delta function:
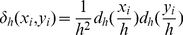
(3)(*x_i_,y_i_*) denote the *i* th Lagrangian point of an elastic element in the bundle. Each of the one-dimensional delta functions has the form
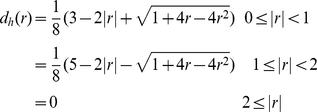
(4)


These forces are incorporated into the fluid solver and the flow solution is advanced using Chorin's projection method [17]. Finally the no-slip condition, Equation (1e), is applied to update the position of the hair bundle. Velocity boundary conditions are given on each of the four sides of the computational fluid domain rectangle: for the bottom we use the horizontal and vertical motions measured by Fridberger et al [7]; for the top we use the previous vertical motion, but set the horizontal motion to zero; on the sides we use the analytical solution given Carslaw & Jaeger [18] for the flow in a channel, with no bundle, driven by the oscillatory motion of one wall.

## Supporting Information

Figure S1 **Dynamic calibration of model.** Using the orbital motion of the lower boundary, the reticular lamina, measured in [7] as input to the calculation, the computed motion of the inner hair cell bundle agrees with the measured amplitude. The slight difference phase between the computed and measured phase could be due to differences in phase of the individual rows.
(TIFF)

Figure S2 **Tip link stretching as a function of phase of reticular lamina motion when no vertical acceleration is present.** The lower tip link does not develop significant tension thereby reducing the sensitivity and coherence of the bundle.
(TIFF)

Table S1 **Model parameters.**
(DOC)

Video S1 **Motion of individual rows of stereocilia in response to acoustical input.**
(MOV)
