# Supplementary figures and images for: Simulation of the Response of the Inner Hair Cell Stereocilia Bundle to an Acoustical Stimulus
Source: PLoS One. 2011 Mar 31;6(3):e18161. doi: 10.1371/journal.pone.0018161 (PMC3069064; doi:10.1371/journal.pone.0018161)

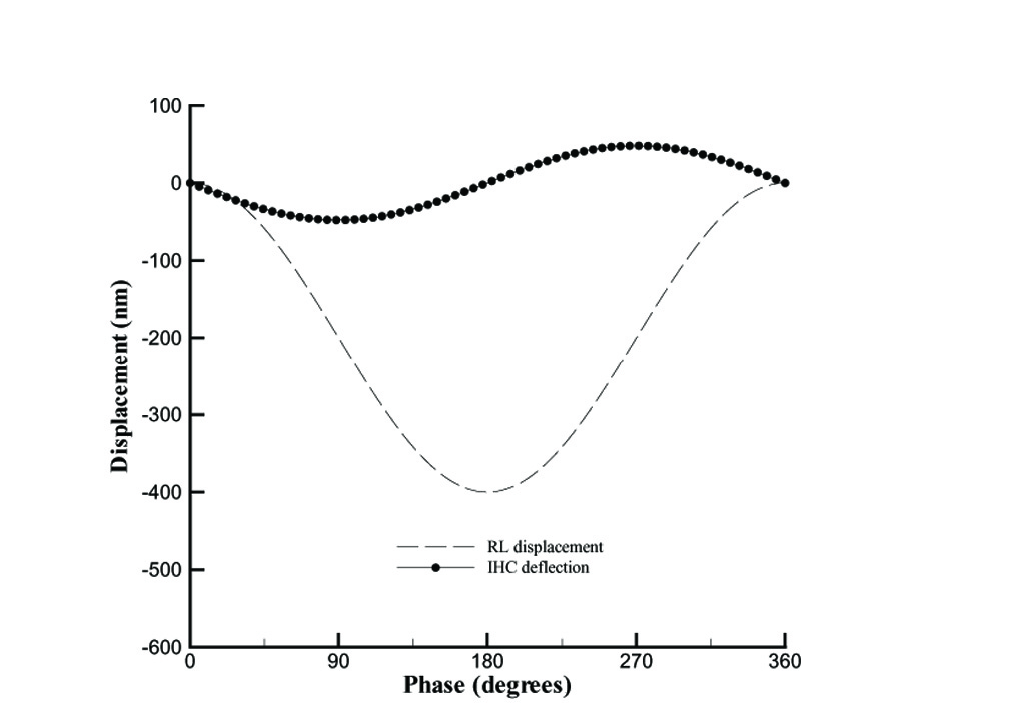

Supplement: Figure S1 — Dynamic calibration of model. Using the orbital motion of the lower boundary, the reticular lamina, measured in [7] as input to the calculation, the computed motion of the inner hair cell bundle agrees with the measured amplitude. The slight difference phase between the computed and measured phase could be due to differences in phase of the individual rows. (TIFF) [file pone.0018161.s001.tif]

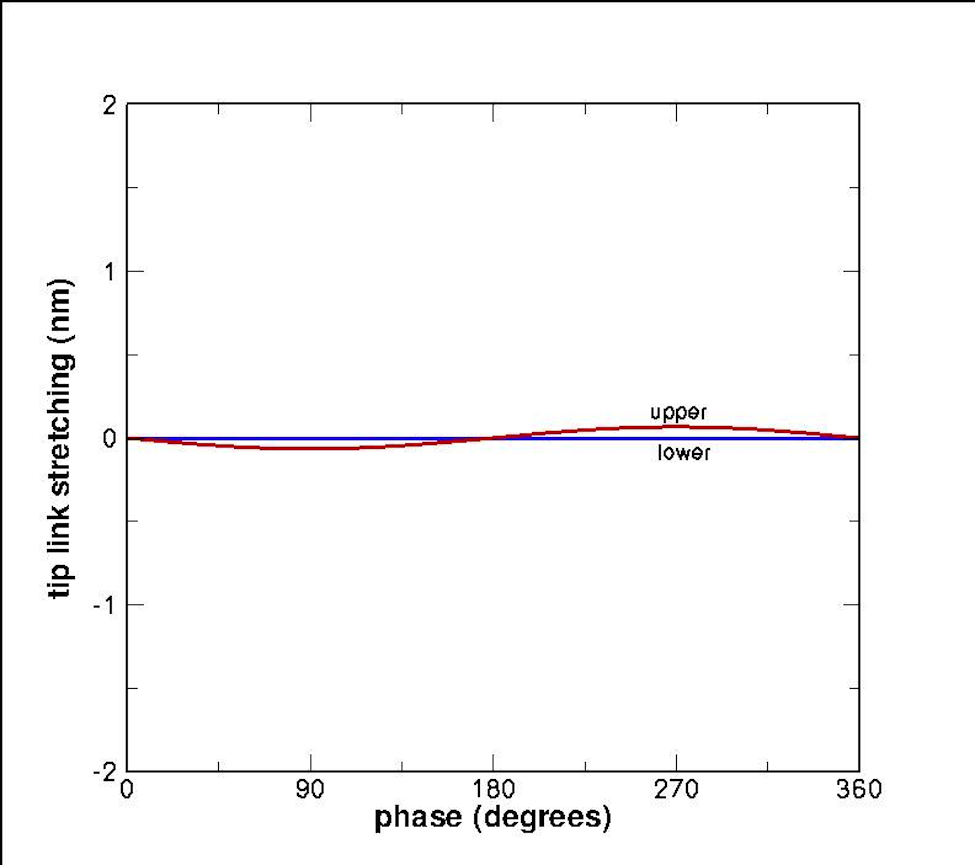

Supplement: Figure S2 — Tip link stretching as a function of phase of reticular lamina motion when no vertical acceleration is present. The lower tip link does not develop significant tension thereby reducing the sensitivity and coherence of the bundle. (TIFF) [file pone.0018161.s002.tif]
