# Supplementary material for: Simulation of the Response of the Inner Hair Cell Stereocilia Bundle to an Acoustical Stimulus
Source: PLoS One. 2011 Mar 31;6(3):e18161. doi: 10.1371/journal.pone.0018161 (PMC3069064; doi:10.1371/journal.pone.0018161)
Supplement: Table S1 — Model parameters. (DOC) [file pone.0018161.s003.doc]

Table S1 Model Parameters

| parameter | value |
| --- | --- |
| fluid density ρ         | 1.0 x 10^3^ kg/m^3^ |
| fluid viscosity μ        | 0.659 x 10^-3^   Pa-s |
| fluid channel height | 5.0 μm |
| fluid channel length | 20.0 μm |
| stereocilia lengths short | 1.5 μm |
| middle | 3.0  μm |
| tall | 4.5 μm |
| stereocilia diameters top | 240 nm |
| d bottom | 30 nm |
| tip link length | 170 nm |
| lower tip link angle | 66^o^ |
| upper tip link angle | 55^o^ |
| gate length | 5 nm |
| gate tension threshold | 26.5 pN |
| link stiffness | 5.4 x 10^-4^ N/m |

**Supplementary Figure Captions**

**Figure S1 Dynamic calibration of model.** Using the orbital motion of the lower boundary, the reticular lamina, measured in (7) as input to the calculation, the computed motion of the inner hair cell bundle agrees with the measured amplitude. The slight difference phase between the computed and measured phase could be due to differences in phase of the individual rows.

**Figure S2 Tip link stretching as a function of phase of reticular lamina motion when no vertical acceleration is present.** The lower tip link does not develop significant tension thereby reducing the sensitivity and coherence of the bundle.
